# Sb_2_Se_3_ assembling Sb_2_O_3_@ attapulgite as an emerging composites for catalytic hydrogenation of *p*-nitrophenol

**DOI:** 10.1038/s41598-017-03281-z

**Published:** 2017-06-12

**Authors:** Lin Tan, Aidong Tang, Yue Zou, Mei Long, Yi Zhang, Jin Ouyang, Jing Chen

**Affiliations:** 1 School of Chemistry and Chemical Engineering, Central South University, Changsha, 410083 China; 2 Centre for Mineral Materials, School of Minerals Processing and Bioengineering, Central South University, Changsha, 410083 China; 3 Hunan Key Laboratory of Mineral Materials and Application, Central South University, Changsha, 410083 China; 4 Key Laboratory of Palygorskite Science and Applied Technology of Jiangsu Province, Huaiyin Institute of Technology, Huaian, 223003 China

## Abstract

The construction and application of a new type of composite material are achieved more and more attention. However, expected Sb_2_Se_3_/attapulgite composites aim to use the low price, and high adsorption of attapulgite in assembling Sb_2_Se_3_ is quite difficult to be acquired by a facile and benign environmental hydrothermal method. In this manuscript, we developed a new way for preparation of an emerging composite by means of Sb_2_O_3_ as a media linking Sb_2_Se_3_ and attapulgite together, and finally won an emerging composite Sb_2_Se_3_/Sb_2_O_3_@attapulgite, which presented an excellent catalytic properties for catalytic hydrogenation of *p*-nitrophenol. It was noted that the Sb_2_Se_3_/Sb_2_O_3_@attapulgite composites exhibited a high conversion rate for the hydrogenation of *p*-nitrophenol that was up to 90.7% within 15 min, which was far more than the 61.5% of Sb_2_Se_3_ sample. The excellent catalytic performance was attributed to the highly dispersion Sb_2_Se_3_ microbelts and Sb_2_Se_3_@Sb_2_O_3_@attapulgite rods, which would improve the adsorption of the reactant species and facility electronic transfer process of the catalytic hydrogenation of p-nitrophenol.

## Introduction

General one-dimensional (1D) nanomaterials such as carbon nanotubes, metal or semiconducting nanowires and their composites which have been widely applied in catalysis^1–5^, energy conversion and storage devices^6–12^, gas sensors^13, 14^ etc. More and more emerging composites will offer a perspective for new entries into novel structures and high technology applications^15–23^. Sb_2_Se_3_, one of a typical 1D nanostructured material in the group V–VI binary semiconductors, has attracted considerable attention due to its unique photovoltaic^24^, electrochemical properties^25^ and efficient catalytic performance^26^. The synthesis of Sb_2_Se_3_ using facile hydrothermal or solvothermal method has been extensively studied^25–29^. A large number of advanced techniques have been developed to fabricate one-dimensional (1D) nanostructured composites with well-controlled morphology and chemical composition. Among these methods, hydrothermal method seems to be the simplest and most versatile technique capable of generating 1D nanostructures (mainly nanorods, nanoflakes)^30, 31^. However, application of Sb_2_Se_3_ was limited since relatively low catalytic activity of rod-like bulk Sb_2_Se_3_ with small specific area and easy aggregation of Sb_2_Se_3_ nanoparticles with good property but slightly poor stability. Therefore, one of method for inhibiting aggregation and enhancing stability was to assemble the functional material onto another support material, such as carbon materials^32, 33^, metal oxides^34, 35^, natural clay mineral^36, 37^, and so on. However, the synthesis of Sb_2_Se_3_ nanocomposites using facile and benign environment hydrothermal method has not been extensively studied. In particular, in a Sb_2_Se_3_ structures unit, (Sb_4_Se_6_)_n_ ribbons grow along the (001) direction through covalent Sb–Se bonds, while hold with adjacent (Sb_4_Se_6_)_n_ ribbons by van der Waals interactions^24, 28^. Therefore, Sb_2_Se_3_ is easy to form a rod like morphology and difficult to anchor onto the surface of support. Thus, to develop a facile process for inhibiting the Sb_2_Se_3_ aggregation still remains a huge challenge.

Attapulgite also called palygorskite (Pal), is a species of 1D fiber-shape hydrated magnesium aluminum silicate with the theoretical formula (Mg,Al,Fe)_5_Si_8_O_20_(OH)_2_·4H_2_O^38^. The attapulgite fiber is consisted of two layers of tetrahedral silica and connected by a Al or Mg octahedral configuration. It is widely used in adsorbents, catalysts and catalyst supports due to the 1D fiber shape, high specific surface area, nontoxicity, low cost and numerous hydroxyl groups^39–42^. However, the attapulgite with nanoscale fiber-like morphology is always aggregated to bulk crystal bundles in raw attapulgite, which would limit the utilization of the unique property of attapulgite^43^. So, the disaggregation of attapulgite crystal bundles plays an important role in the fabrication of attapulgite composites. Recently, Wang’s groups have discussed the method of dispersion of crystal bundles or aggregates of natural attapulgite^43^. The disaggregated method of the aggregated attapulgite rod crystals includes ball grinding^44^, irradiation^45^, high-speed shearing^46^ and ultrasonication^47^ etc. Compare to other method, ultrasonication could scatter the materials through controlling the size of nanoscale attapulgite by altering the ultrasonication parameter effectively. By far, the fabrication of Sb_2_Se_3_/attapulgite composite has not been explored in detail. The possibility of preparing functional nanomaterials via selectively assembling SbO^+^ on the surface of natural clay templates is expected to provide a range of new opportunities.

**Figure 1:**
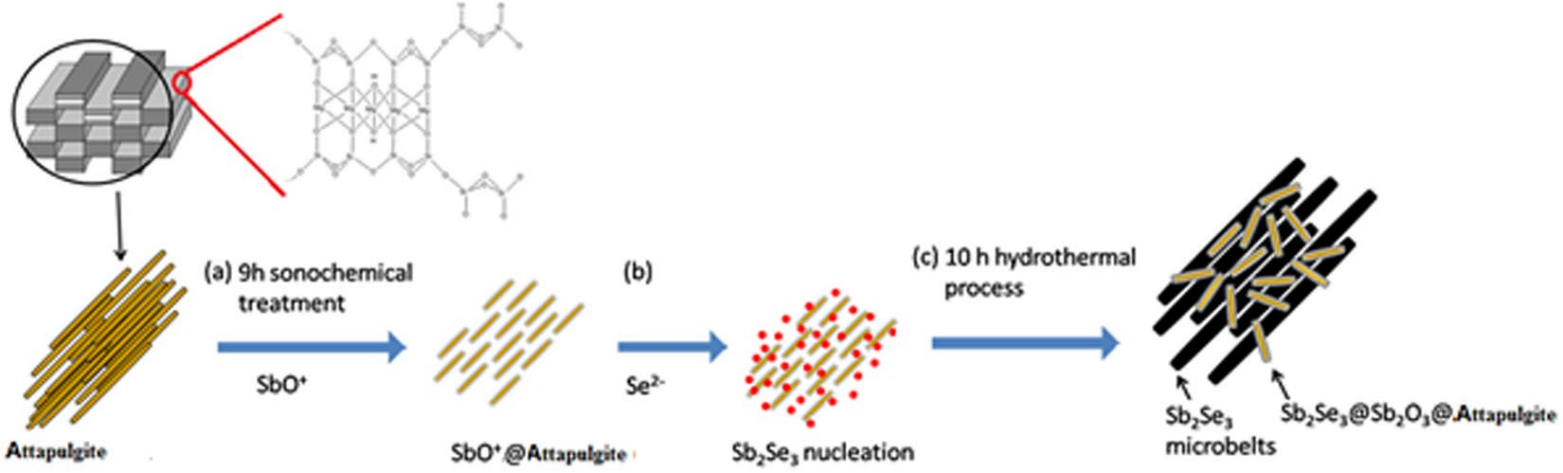
Schematic diagram of a synthesis strategy of the Sb_2_Se_3_/Sb_2_O_3_@attapulgite composites.

In this study, as Fig. 1 illustrated, we proposed a strategy to disaggregate crystal bundles of attapulgite by taking full advantage of the excellent adsorption of the attapulgite thank to the electrostatic interaction between the negative charge of Si-OH and the SbO^+^ ions released from hydrolysis of antimony potassium tartrate. Meanwhile, during the ultrasonic disaggregation process of attapulgite crystal bundles, the SbO^+^ ions were greatly adsorbed onto the surface of attapulgite.

Namely, small rod-like of attapulgite nanoparticles used as a template for assembling SbO^+^ *in situ*. Thus well-dispersed SbO^+^@attapulgite nanorods through long time sonochemical pretreatment were obtained at first. Then Se^﻿2﻿^^−^ ions produced by a reductant NaBH_4_ reacting with selenium powder were introduced to the precursor to form the Sb_2_Se_3_ initial nuclei. Finally an emerging composite was obtained with a long belt-like Sb_2_Se_3_ and small rod-like Sb_2_O_3_@attapulgite by a facile hydrothermal method. The microstructures and morphologies of the nanocomposites were investigated. Moreover, the interfaces between Sb_2_Se_3_, Sb_2_O_3_ and attapulgite were explored and discussed. In addition, the catalytic performance of nanocomposites were tested by the catalytic hydrogenation of p-nitrophenol (p-NP) to p-aminophenol (p-AP) and the synergistic effects between Sb_2_Se_3_ and Sb_2_O_3_@attapulgite were also investigated.

## Results and Discussion

**Figure 2:**
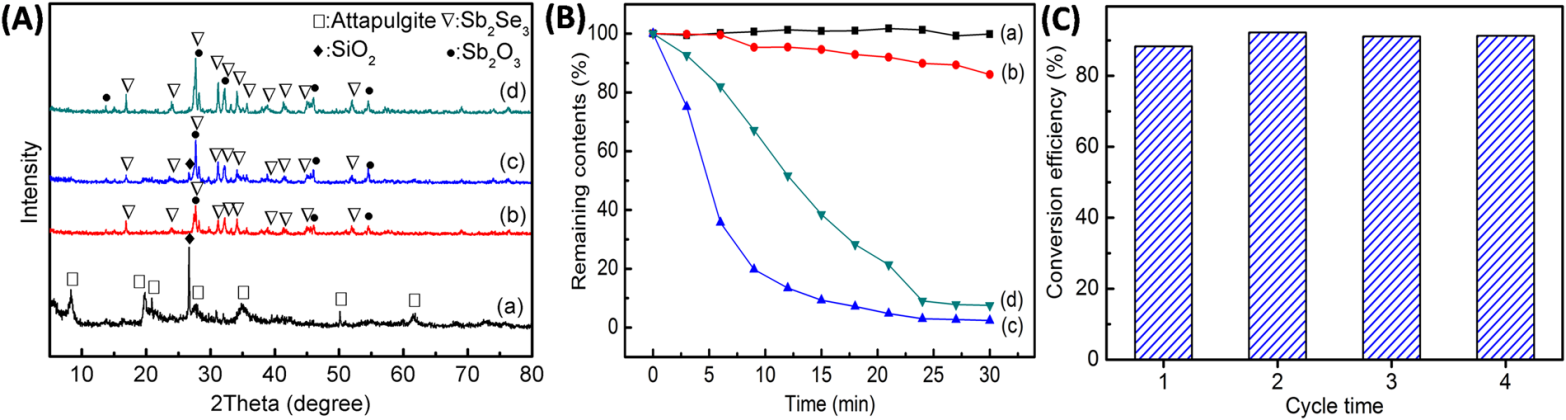
(**A**) XRD patterns of (a) pure attapulgite, (b) Sb_2_Se_3_ sample, (c) Sb_2_Se_3_-Sb_2_O_3_-attapulgite and (d) Sb_2_Se_3_/Sb_2_O_3_@attapulgite; (**B**) Plots for the catalytic reduction of p-NP by (a) pure attapulgite, (b) Sb_2_Se_3_-Sb_2_O_3_-attapulgite, (c) Sb_2_Se_3_/Sb_2_O_3_@attapulgite and (d) Sb_2_Se_3_ sample and (**C**) the recyclability of the as-prepared Sb_2_Se_3_/Sb_2_O_3_@attapulgite for catalytic hydrogenation of p-NP.

XRD patterns of pure attapulgite, Sb_2_Se_3_-Sb_2_O_3_-attapulgite, Sb_2_Se_3_/Sb_2_O_3_@attapulgite composites and the pure Sb_2_Se_3_ sample were given in Fig. 2A. The XRD pattern in Fig. 2A(a) of raw attapulgite was in accordance with the attapulgite (PDF No. 29-0855). Meanwhile, the high intensity diffraction peak at 26.7° was attributed to the quartz^48^. For the Sb_2_Se_3_ sample in Fig. 2A(b), the peaks at 27.5°, 31.2°, 32.3° and 34.2° were indexed to (230), (221), (301) and (240) crystal planes of the orthorhombic Sb_2_Se_3_ phase respectively (PDF No. 15-0861). And the peaks at 27.7°, 32.2°, 46.0° and 54.6° were attributed to the senarmontite of Sb_2_O_3_ (PDF No. 43-1071).

For Sb_2_Se_3_-Sb_2_O_3_-attapulgite in Fig. 2A(c), apart from the characteristic peaks of Sb_2_Se_3_ and Sb_2_O_3_, the characteristic peaks of the quartz could be also included, but no peak was attributed to attapulgite. Moreover, the peak intensities of Sb_2_Se_3_ in Fig. 2A(c) was partly lower than that of in Fig. 2A(b). The above results implied that the Sb_2_Se_3_ and Sb_2_O_3_ have produced on the attapulgite surface. However, the diffraction peak of the quartz at 26.7° still remained, indicating that the Sb_2_Se_3_ and Sb_2_O_3_ particle only partly coated onto the attapulgite surface. In Fig. 2A(d), related reflection peaks also could attribute to Sb_2_Se_3_ or Sb_2_O_3_ but the reflection peaks of attapulgite and quartz were disappeared. This phenomenon implied that the Sb_2_O_3_ particles have covered the attapulgite surface entirely via sonochemical pretreatment for a long time. Observe carefully, compare to Sb_2_Se_3_ sample and Sb_2_Se_3_-Sb_2_O_3_-attapulgite sample, the intensities of the Sb_2_Se_3_ in Sb_2_Se_3_/Sb_2_O_3_@attapulgite sample are clearly higher, indicating that well-dispersed Sb_2_O_3_@attapulgite might be helpful to the growth of Sb_2_Se_3_ particles.

The catalytic performance of as-synthesized samples was tested and the concentration of p-NP ions was monitored through visible spectrophotometer. Figure 2B showed the remaining concentration of the p-NP ions after adding different samples. It was noted that the absorbance were increased insignificant when added pure attapulgite within 30 min, which meant that the raw attapulgite had no contribution to the catalytic activity. For the Sb_2_Se_3_-Sb_2_O_3_-attapulgite sample, the conversion rates of p-NP were just 14% within 30 min. But when the same amounts of precursors were pretreated by 9 h of sonochemical pretreatment, the conversion of p-NP could exceed 90% within 15 min for the Sb_2_Se_3_/Sb_2_O_3_@attapulgite while the same conversion of p-NP needs 24 min for the Sb_2_Se_3_ sample.

More catalytic activity of the composites with different attapulgite added amount was observed in Fig. S1. According to Fig. S1, the p-NP conversion rate at 30 min of composites with sonochemical pretreatment was all far higher than that of the composites at the same attapulgite mass contents without sonochemical pretreatment. For comparison, the catalytic performance of Sb_2_O_3_ as single component was also considered shown in Fig. S2. Sb_2_O_3_ presents slowly catalytic activity for catalytic hydrogenation of p-NP at the initial catalytic process. The p-NP conversion rate at 30 min is only 17.5%, indicating that the excellent catalytic performance of Sb_2_Se_3_/Sb_2_O_3_@attapulgite composites is attributed to the synergistic effect among Sb_2_Se_3_, Sb_2_O_3_ and attapulgite. The catalytic mechanism needs further research.

Reusability was also very important for the application of a catalyst. Herein, the reusability of the as-prepared Sb_2_Se_3_/Sb_2_O_3_@attapulgite composites was evaluated by detecting the catalytic reduction efficiency within 15 min of four repeated experiments and the results were shown in Fig. 2C. It was observed that the conversion efficiency of p-NP could reach up to 90% within 15 min after the 4th recycle. The results indicated that the as-prepared Sb_2_Se_3_/Sb_2_O_3_@attapulgite could be repeatedly used and provided stable performance. In summary, the Sb_2_Se_3_/Sb_2_O_3_@attapulgite composite showed outstanding catalytic performance, relatively low cost will present a good potential in practical applications.

**Figure 3:**
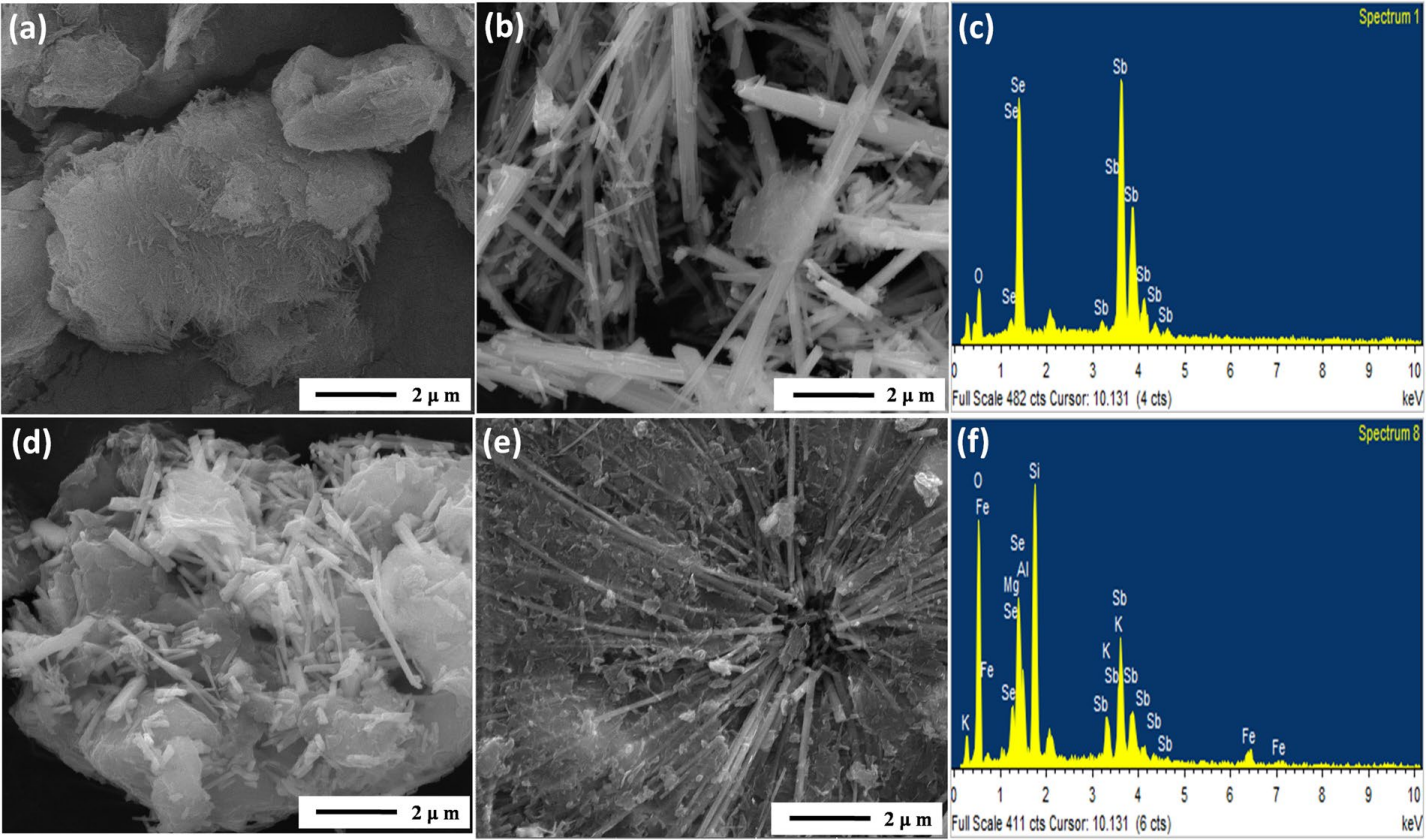
SEM images of (**a**) raw attapulgite, (**b**) Sb_2_Se_3_ sample, (**d**) Sb_2_Se_3_-Sb_2_O_3_-attapulgite, (**e**) Sb_2_Se_3_/Sb_2_O_3_@attapulgite composites and EDS spectra of (**c**) Sb_2_Se_3_ sample and (**f**) Sb_2_Se_3_/Sb_2_O_3_@attapulgite.

To reveal the morphology of the as-synthesized sample and demonstrate the existing states of Sb_2_Se_3_, Sb_2_O_3_ and attapulgite, SEM and EDS were detected and showed on Fig. 3, Fig. S3 and Fig. S4. For the raw attapulgite (Fig. 3a and S3a), many small bundles with 20–50 nm in diameters were aggregated into fibrous bunches and sheet-like layers owing to the strong interaction between small bundles crystals^49^. In the Fig. 3b, it could find that the Sb_2_Se_3_ sample was composed of abundant irregular belt shape structures with diameters ranging from 100 to 700 nm and lengths of several micrometres. However, according to the high magnification SEM images of Sb_2_Se_3_ sample (Fig. S3b), the large size microbelts were consisted of the severe aggregation of several Sb_2_Se_3_ nanobelts. After Sb_2_Se_3_ and Sb_2_O_3_ particles loaded on attapulgite surface directly, it could be observed that the original fibrous bunches of attapulgite have covered by densely several irregular structures and numerous rod shape particles (Fig. 3d). Observe carefully, the rod like composites with diameters about 200 nm and length about 1 μm were distributed on the several irregular structures surface. The results revealed that Sb_2_Se_3_ and Sb_2_O_3_ were combined not well with attapulgite in the hydrothermal process. But when the precursor contained antimony potassium tartrate and attapulgite was pretreated by 9 h of sonochemical treatment, several uniform microbelts with a diameters of 100–200 nm could be found in Fig. 3e. Interestingly, some dispersed rod structure displayed in Fig. S4b were stacked approximately parallelly on the surface of microbelts which would expose more catalytic reaction sites. To clearly manifest the size of microbelts, the size distribution diagram of Sb_2_Se_3_ sample and Sb_2_Se_3_/Sb_2_O_3_@attapulgite composites were displayed in Fig. S4c and d respectively. According to the statistics results, the average width of the microbelts in Sb_2_Se_3_/Sb_2_O_3_@attapulgite was 147.17 nm, which was lower than that of 173.66 nm in Sb_2_Se_3_ sample. This may be due to highly dispersed bundles of attapulgite effectively inhibited the aggregation of Sb_2_Se_3_ microbelts.

The composition of Sb_2_Se_3_ sample and Sb_2_Se_3_/Sb_2_O_3_@attapulgite were detected by energy dispersive spectroscopy (EDS) and the results as Fig. 3c and f displayed respectively. According to the Fig. 3c, three elements of Sb, Se and O were found which indicated the Sb_2_Se_3_ was fabricated. As Fig. 3f displayed, the symbol of O, Si, Al, Mg, K and Fe were assigned to the attapulgite, while Sb, Se and other parts of O originated from Sb_2_Se_3_ and Sb_2_O_3_. Moreover, the contents of Se and Sb in Sb_2_Se_3_-Sb_2_O_3_-attapulgite and Sb_2_Se_3_/Sb_2_O_3_@attapulgite were confirmed by ICP method. Among them, the mass contents of Sb and Se were 17.3% and 9.39% respectively for Sb_2_Se_3_-Sb_2_O_3_-attapulgite composites. When the precursor was pretreated by 9 h of sonochemical pretreatment, the Sb content almost kept constant (17.5%), while the Se content decreased to 5.99%. The results demonstrated that more SbO^+^ converted into Sb_2_O_3_ under 9 h of sonochemical pretreatment. The above results and XRD results demonstrated that Sb_2_Se_3_/Sb_2_O_3_@attapulgite nanocomposites were obtained. More importantly, the long time sonochemical pretreatment could promote the exfoliation of attapulgite crystal bundles, which was further promoted the Sb_2_Se_3_ growth and combined with Sb_2_O_3_@attapulgite.

**Figure 4:**
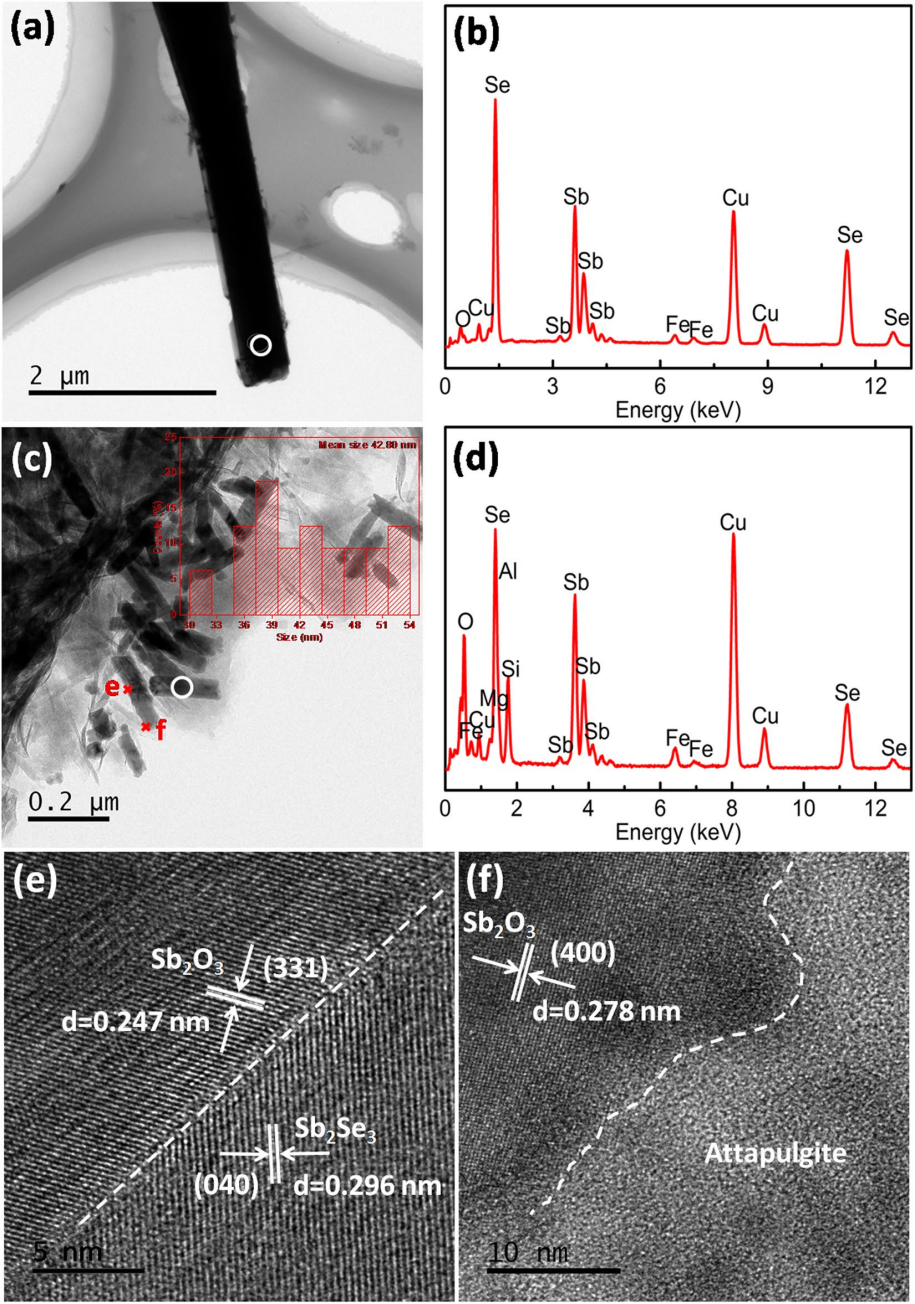
(**a,c**) TEM images, the inset showed the corresponding size distribution diagram, (**b,d**) EDS and (**e,f**) HRTEM images of Sb_2_Se_3_/Sb_2_O_3_@attapulgite corresponded to the indicating region in (**c**).

To further investigate morphology, structure and interfaces among Sb_2_Se_3_, Sb_2_O_3_ and attapulgite, the TEM images of Sb_2_Se_3_/Sb_2_O_3_@attapulgite were presented in Fig. 4. The EDS result of the signal region on the microbelt in Fig. 4a was given in Fig. 4b. The element Sb and Se could be found in the EDS spectrum, indicated that the microbelt was composed of Sb_2_Se_3_. Meanwhile, the element O could also be observed in the EDS spectrum which indicated that tiny amounts of Sb_2_O_3_ attached to Sb_2_Se_3_ microobelts. As can be found in Fig. 4c, attapulgite rods were homogeneously covered by the nanoparticles. In addition, the nanorods with 42.8 nm in average diameter and several hundred nanometers in length touched each other directly or connected by the single rods, but no larger agglomerates could be observed. EDS analysis of the rod was showed in Fig. 4d. The element O, Si, Al, Mg, Fe, Sb and Se could be found in the EDS spectrum, demonstrated that the nanorods were composed of Sb_2_Se_3_, Sb_2_O_3_ and attapulgite. In order to ascertain the interfaces among Sb_2_O_3_ particles, Sb_2_Se_3_ particles and attapulgite, the HRTEM images of the nanorods were detected and showed in Fig. 4e and 4f. The interplanar spacings of about 0.247 nm and 0.296 nm in Fig. 4e, which were corresponded to the (331) plane of Sb_2_O_3_ (PDF No.43-1071) and (040) plane of Sb_2_Se_3_ (PDF No. 15-0861) respectively. The white dashed line in Fig. 4f showed the neighboring of attapulgite and Sb_2_O_3_ particles, while the interplanar distance of 0.278 nm was corresponded to the (400) plane of Sb_2_O_3_ (PDF No. 43-1071). The above HRTEM images clearly revealed the interface between attapulgite, Sb_2_O_3_ and Sb_2_Se_3_ particles. On the basis of TEM demonstration, the Sb_2_Se_3_/Sb_2_O_3_@attapulgite composites were constructed by independent Sb_2_Se_3_ microbelts and Sb_2_Se_3_@Sb_2_O_3_@attapulgite rod shape architectures.

**Figure 5:**
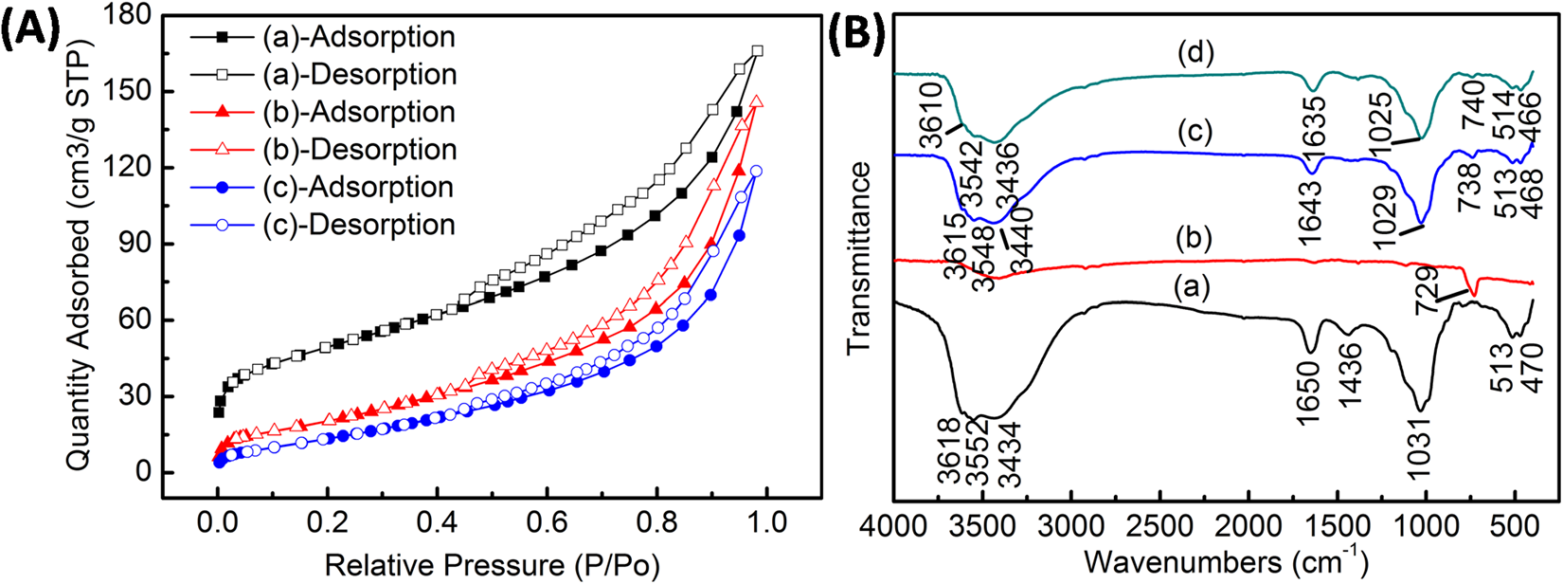
(**A**) Nitrogen adsorption–desorption isotherm of (a) raw attapulgite, (b) Sb_2_Se_3_-Sb_2_O_3_-attapulgite and (c) Sb_2_Se_3_/Sb_2_O_3_@attapulgite; (**B**) FTIR spectra of the samples: (a) raw attapulgite, (b) Sb_2_Se_3_sample, (c) Sb_2_Se_3_-Sb_2_O_3_-attapulgite and (d) Sb_2_Se_3_/Sb_2_O_3_@attapulgite.

The nitrogen adsorption–desorption isotherms were shown in Fig. 5A. All the isotherms showed in Fig. 5A were type II isotherms according to the IUPAC classifications. When the P/P_0_ > 0.4, the hysteresis loop appeared which demonstrated some degree of mesoporosity in the samples. The structural characteristics of samples were shown in Table 1. The BET specific surface area and micropore area of the raw attapulgite were 175.589 m^2^ g^−1^ and 26.806 m^2^ g^−1^ respectively. But when the attapulgite surface was coated by Sb_2_Se_3_ and Sb_2_O_3_ particles, the BET specific surface area and micropore area of the Sb_2_Se_3_-Sb_2_O_3_-attapulgite were just 86.996 m^2^ g^−1^ and 0.000 m^2^ g^−1^ respectively. The results were mainly attributed to the Sb_2_Se_3_ and Sb_2_O_3_ particles coated on the surface of attapulgite, resulting in the amounts of voids and pores decrease and similar effect had been reported by Li *et al*.^50^. With 9 h sonochemical pretreatment to the precursor, the BET specific surface area further decreased to 65.073 m^2^ g^−1^. According to the SEM results, the further decreased in the specific surface area may be due to the larger size of Sb_2_Se_3_ microbelts and the Sb_2_Se_3_ and Sb_2_O_3_ particles anchored on the surface of attapulgite which could further block of voids and pores. Overall, the Sb_2_Se_3_/Sb_2_O_3_@attapulgite composite presents minimum specific surface area, indicating that the large specific surface area and micropore area of attapulgite greatly decrease due to the product of hydrolysis coating on the surface of attapulgite after sonochemical treatment. Therefore, it is reasonable to propose that Sb_2_Se_3_ may be linked with attapulgite by the product of hydrolysis and crystallization Sb_2_O_3_ after sonochemical treatment and hydrothermal method.

**Table 1:** Structural characteristics of (a) raw attapulgite, (b) Sb_2_Se_3_-Sb_2_O_3_-attapulgite nanocomposites and (c) Sb_2_Se_3_/Sb_2_O_3_@attapulgite nanocomposites.

Samples	S_BET_ (m^2^/g)	S_micro_ (m^2^/g)	S_ext_ (m^2^/g)	V_micro_ (cm^3^/g)	V_total_ (cm^3^/g)	PZ (nm)
(a)	175.589	26.806	148.783	0.012	0.2567	5.847
(b)	86.996	0.000	86.996	0.000	0.2252	10.355
(c)	65.073	0.000	65.073	0.000	0.1835	11.282

In order to further investigate the interaction among Sb_2_Se_3_, Sb_2_O_3_ and attapulgite, the FTIR spectra of attapulgite, Sb_2_Se_3_ sample, Sb_2_Se_3_-Sb_2_O_3_-attapulgite and Sb_2_Se_3_/Sb_2_O_3_@attapulgite were characterized and showed in Fig. 5B. As the FTIR spectrum of attapulgite shown in Fig. 5B(a), the spectrum in the range between 3700 cm^−1^ and 3200 cm^−1^ was due to the structure O-H stretching vibrations^49^. The bands at 470 cm^−1^ and 513 cm^−1^ were due to the Si-O-Si bonds bending vibration while the band around 1031 cm^−1^ was assigned to the Si-O stretching vibration^48, 51^. Meanwhile, the peak at 1650 cm^−1^ and 1436 cm^−1^ were assigneded to the O-H bending vibration and carbonate impurities respectively^41, 52^. It was noted that all absorption bands of attapulgite in Fig. 5B from (a) to (c) and (d) shifted towards lower wavenumber. However, for Sb_2_Se_3_ sample, the peak at 729 cm^−1^ shown in Fig. 5B(b) was assigned to stretching vibration of Sb-O band of Sb_2_O_3_^53^. It was noted that Sb-O bands from (a) to (c) and (d) shifted towards higher wavenumber. On the other hand, the intensities of these bands also were declining sharply by making comparison with the initial attapulgite. After treated with 9 h of sonochemical pretreatment, the shifted wavenumbers and the decreased intensities could also be found in Sb_2_Se_3_/Sb_2_O_3_@attapulgite in Fig. 5B(d). A possible explanation for these observations was the strong interaction through Si-O-Sb and O-Sb-Se bond among the attapulgite, Sb_2_O_3_ and Sb_2_Se_3_ which lead to the shifts of wavenumbers and the decrease of intensity^54, 55^. More importantly, the sonochemical pretreatment further enhanced this interaction among Sb_2_Se_3_, Sb_2_O_3_ and attapulgite. In a word, the FTIR results implied that the Sb_2_Se_3_ combined with attapulgite through Sb_2_O_3_ and formed the Sb_2_Se_3_@Sb_2_O_3_@attapulgite composite.

To investigate the optical properties of the composites, the UV-Vis diffuse reflectance spectra of Sb_2_Se_3_-Sb_2_O_3_-attapulgite and Sb_2_Se_3_/Sb_2_O_3_@attapulgite composites were tested and the results were displayed in Fig. S5. Among them, Sb_2_Se_3_-Sb_2_O_3_-attapulgite and Sb_2_Se_3_/Sb_2_O_3_@attapulgite showed a broad absorption band between 250 and 800 nm (Fig. S5A). In addition, the band gap energy of above two samples were estimated by Tauc’s formula and the results were shown in Fig. S5B. According to the (αhv)^2^-(hv) plot, the band gap energies were 1.70 eV and 1.34 eV for Sb_2_Se_3_-Sb_2_O_3_-attapulgite and Sb_2_Se_3_/Sb_2_O_3_@attapulgite respectively. As previous similar literature reported, due to the quantum confinement effect of nanoparticles, the band gap energy of semiconductor was increased with the nanoparticles size decreased^56^. Therefore, the Sb_2_Se_3_/Sb_2_O_3_@attapulgite showed well crystallinity which would lead to the direct band gap decrease to 1.34 eV, similar situation was raised by Yang^57^. Herein, due to the sonochemical pretreatment, highly dispersed Sb_2_O_3_@attapulgite rods play a key role in the inhibiting the Sb_2_Se_3_ aggregation and promoting the Sb_2_Se_3_ growth.

**Figure 6:**
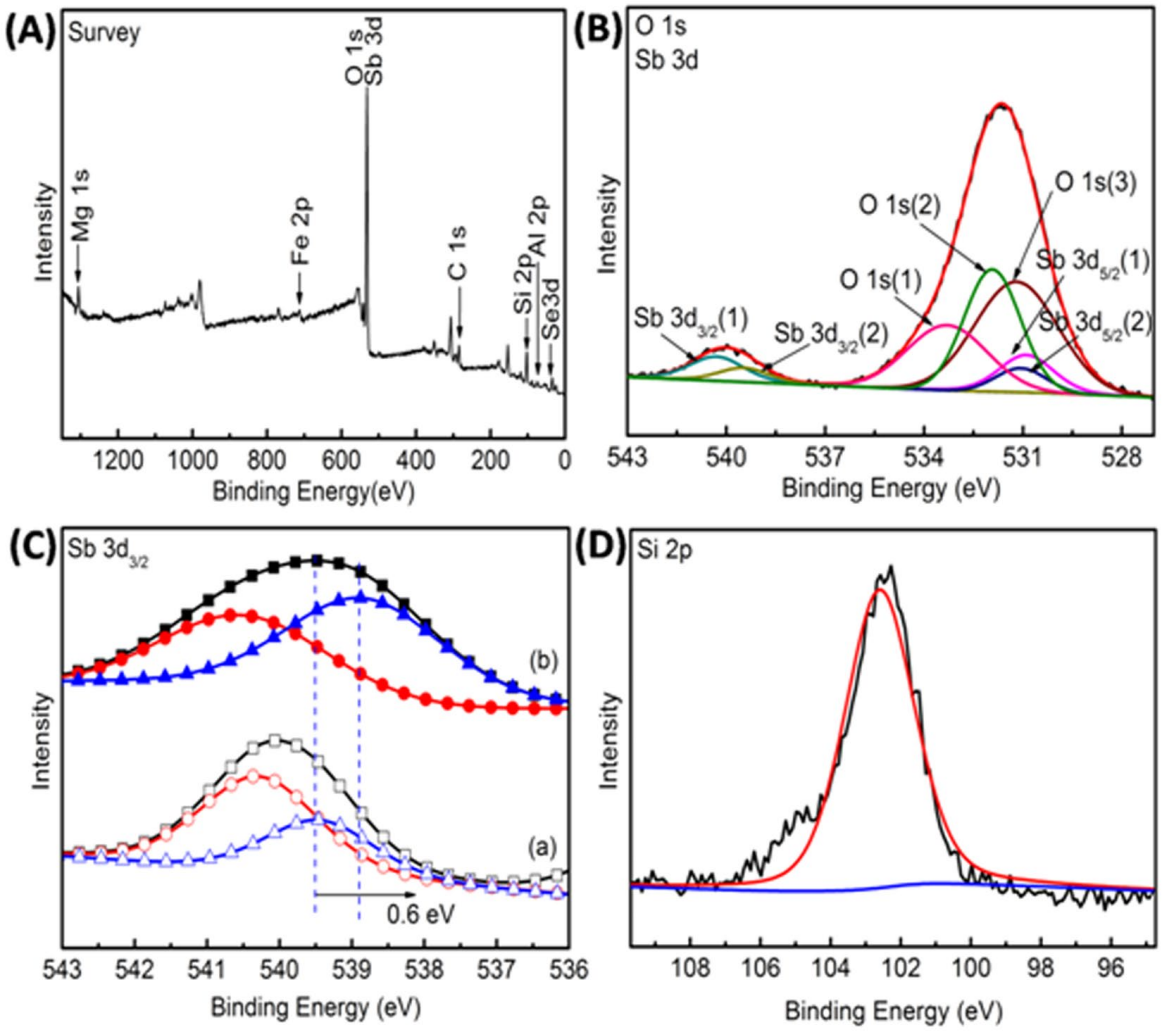
XPS spectra of (**A**) survey, (**B**) O 1s and Sb 3d (**D**) Si 2p region for Sb_2_Se_3_/Sb_2_O_3_@attapulgite and XPS patterns of (**C**) Sb 3d_3/2_ for (a) Sb_2_Se_3_/Sb_2_O_3_@attapulgite and (b) Sb_2_Se_3_ sample.

To investigate the bond environment of the sample of Sb_2_Se_3_/Sb_2_O_3_@attapulgite, XPS test was carried out and the results were presented in Fig. 6. The wide scan survey of Sb_2_Se_3_/Sb_2_O_3_@attapulgite in Fig. 6A showed characteristic peaks of Mg 1s, Fe 2p, O 1s, Sb 3d, C 1s, Si 2p, Al 2p and Se 3d, which demonstrated that Sb_2_Se_3_/Sb_2_O_3_@attapulgite composite was fabricated. Among them, the O 1s peak and Sb 3d peak of the composites were shown in Fig. 6B. The binding energy at 533.3 eV signed O 1s(1) and 531.0 eV signed O 1s(3) were assigned to the oxygen of adsorbed water and hydroxyl groups of the attapulgite respectively^58^. The peak at 531.7 eV was assigned to oxygen of Sb_2_O_3_^59^. Meanwhile, two peaks centered around 540.2 eV (Sb 3d_3/2_(1)) and 530.8 eV (Sb 3d_5/2_(1)) were due to the Sb 3d_3/2_ and Sb 3d_5/2_ of Sb_2_O_3_ respectively^60^. Observe carefully, the two peaks at 539.5 eV (Sb 3d_3/2_(2)) and 531.1 eV (Sb 3d_5/2_(2)) attributed to Sb 3d_3/2_ and Sb 3d_5/2_ of Sb_2_Se_3_ respectively which was higher than the previous literature recorded of 537.9 eV and 529.5 eV respectively^27^. The probable explanation was that the Sb^3+^ in Sb_2_Se_3_ state was affected by the Sb_2_O_3_ and attapulgite.

To further reveal the bond environment of the Sb_2_Se_3_/Sb_2_O_3_@attapulgite, the Sb 3d_3/2_ for Sb_2_Se_3_/Sb_2_O_3_@attapulgite and Sb_2_Se_3_ sample were given in Fig. 6C. The binding energy of Sb 3d_3/2_ peak in Sb_2_Se_3_/Sb_2_O_3_@attapulgite was also higher than the binding energy of Sb 3d_3/2_ peak in Sb_2_Se_3_ sample. Herein, when the Sb atom of Sb_2_Se_3_ was linked to Sb_2_O_3_ through O-Sb-Se bond, due to the electron withdrawing effect between oxygen and antimony (electronegativity: O 3.44 > Se 2.55), the electrons of Sb_2_Se_3_ would impart to the Sb_2_O_3_ which would lead the Sb 3d_3/2_ peak shifted to a higher binding energy region. Similar situation was also found in Ag-polymethacrylic acid-clay composites^61^ and butadiene-styrene-vinyl pyridine rubber-graphene oxide composites^62^. At the same time, the peak at 102.6 eV in Fig. 6D was attributed to the Si 2p, which was lower than that of 103.0 eV in raw attapulgite^63^. Similarly, due to the electro-negativity of H (2.20) was higher than the Sb (2.05), when the H atom of Si-O-H bond was replaced by Sb and formed the Si-O-Sb bond, the Si 2p binding energy would shift to a lower binding energy region in the same way. In short, the shift of binding energy and the FTIR results both revealed that the Sb_2_Se_3_ interacted with attapulgite through Sb_2_O_3_, which would further enhance the stability of the composites.

### Discussion about the influence of ultrasonic pretreatment and the mechanism of synthesis

Based on above experimental results, long time ultrasonic pretreatment plays a major role for synthesis of Sb_2_Se_3_/Sb_2_O_3_@attapulgite composites. The possible synthesis mechanism of Sb_2_Se_3_/Sb_2_O_3_@attapulgite composites is illustrated in Fig. 1. The process consists three stages. Firstly, the ultrasonic treatment could disperse and modify the clay as previous literature recorded^64^. Herein, the ultrasonic pretreatment for 9 h not only disaggregated attapulgite rods and reduced the particle size, but also facilitated the SbO^+^ ions transfer to the surface and pores of attapulgite under high-pressure shock waves and acoustic vortex microstreaming. With extended time ultrasonic treatment to the precursor, large amount of SbO^+^ ions would interact with the hydroxyl of attapulgite surface. Therefore, SbO^+^ ions were grafted on the surface and pore of attapulgite by electrostatic interaction through Si-O-Sb bond and formed a compact SbO^+^@attapulgite structure.

Secondly, when the Se^2−^ ions were added into the solution, the Se^2−^ ions would react with SbO^+^ and form Sb_2_Se_3_ initial nuclei. According to a previous report, the Sb_2_Se_3_ nuclei tend to grow into one-dimensional structure without other surfactant presence^25^. Herein, large amounts of Sb_2_Se_3_ initial nuclei grew independently into the belts with [001] orientation. Meanwhile, abundant SbO^+^@attapulgite rods could hinder the growth of Sb_2_Se_3_ and part of SbO^+^@attapulgite incorporate with Sb_2_Se_3_ initial nuclei. Finally, during the hydrothermal process for 10 h, the Sb_2_Se_3_ grew into microbelts with 147.17 nm in mean diameter, while Sb_2_Se_3_@Sb_2_O_3_@attapulgite nanorods loaded on the microbelts surface and worked as steric hindrance which could inhibit the Sb_2_Se_3_ microbelts aggregation. In the end, uniform Sb_2_Se_3_ microbelts stacked by Sb_2_Se_3_@Sb_2_O_3_@attapulgite rod were fabricated. Alternatively, if the precursor contained attapulgite and antimony potassium tartrate was not treated by 9 h of sonochemistry treatment, the SbO^+^ ions was only absorbed on the bulk crystal bundles surface of attapulgite, which would lead the Sb_2_Se_3_ and Sb_2_O_3_ particles grew on the aggregation bundles surface and constructed aggregated irregular structure shown as Fig. 3(d). In short, the sonochemical pretreatment not only improve the disaggregation of attapulgite crystal bundles, but also enhance the combination among the Sb_2_Se_3_, Sb_2_O_3_ and attapulgite and finally obtained the high well-dispersed 1D/1D Sb_2_Se_3_/Sb_2_O_3_@attapulgite composites.

The influence of morphologies on electronic properties of Sb_2_Se_3_ was reported in literatures^28^. A possible growth mechanism is proposed to explain the formation of the 1D Sb_2_Se_3_ nanostructures from the viewpoint of crystal structure^28^. By contrast, the difference is this work proposing a novel method to prepare an emerging composite Sb_2_Se_3_/Sb_2_O_3_@attapulgite by means of Sb_2_O_3_ as a media linking Sb_2_Se_3_ and attapulgite together. Therefore, thin and long Sb_2_Se_3_ microbelts were obtained by means of the space steric effect of highly dispersed bundles of attapulgite. Then, which is the most important factor for the improved catalytic properties, morphology change induced by ultrasonic pretreatment or interface structures of Sb_2_Se_3_@Sb_2_O_3_@attapulgite? As Ma^28^ reported that the hydrogen storage performance of Sb_2_Se_3_ nanostructures depending on their size, which clearly explained the morphology-properties relations. We think morphology change induced by ultrasonic pretreatment does play a certain role for the improved catalytic properties. Furthermore, a higher hydrogen storage capacity of Sb_2_Se_3_ also not allow to ignore. According to the previous literature^65, 66^ the p-NP catalytic hydrogenation process usually underwent following procedure based on the Langmuir-Hinshelwood (LH) model: (1) the p-NP ions and hydrogen molecules adsorbed on the catalyst surface; (2) the electrical transferred to p-NP ions through catalyst and p-NP ions was reduced to p-AP; (3) the p-AP dissociated from the catalyst surface. Among them, the step (1) and step (3) was always regarded as fast process due to the constant stirring. Thus, the step (2) that the reduction of p-NP ions to p-AP ions was considered as the rate-determining step. Herein, the well dispersed Sb_2_Se_3_/Sb_2_O_3_@attapulgite could supply more reactive sites and promote the electronic transference and accelerate the reduction of p-NP ions to p-AP ions in step (2). Therefore, we consider the interface structures of Sb_2_Se_3_@Sb_2_O_3_@attapulgite as the most important factor for the improved the catalytic properties.

## Conclusions

In conclusion, we prepared a novel 1D/1D Sb_2_Se_3_/Sb_2_O_3_@attapulgite composites through a facile hydrothermal method. On the basis of the characterization results, the Sb_2_Se_3_/Sb_2_O_3_@attapulgite composites were comprised of rod like Sb_2_Se_3_@Sb_2_O_3_@attapulgite and belts shape Sb_2_Se_3_. Among them, the rod Sb_2_Se_3_@Sb_2_O_3_@attapulgite composites loaded on the surface of Sb_2_Se_3_ microbelts, which could supply more reactive sites for the p-NP catalytic hydrogenation reaction. In addition, the experimental results demonstrated that the long time ultrasonic pretreatment played a key role in the formation of dispersed SbO^+^@attapulgite which could further inhibit the Sb_2_Se_3_ aggregation and promote the fabrication of uniform rod-belts stacks structure. The results of p-NP catalytic hydrogenation showed that the Sb_2_Se_3_/Sb_2_O_3_@attapulgite composites exhibited enhanced efficiency for reduction reaction of p-NP and the catalytic reduction efficiency could reach 90% within 15 min. The Sb_2_Se_3_/Sb_2_O_3_@attapulgite composites showed excellent catalytic hydrogenation performance with comparatively low cost and have potential for application in catalysts. More importantly, this work provides an inspiration to controllabe fabricate composites.

## Methods

### Materials and preparation

Attapulgite was obtained from Xuyi Botu Attapuligite Hi-Tech Development Co, Ltd, Jiangshu, China. Other chemicals were of analytical grade and were used as received, while aqueous solutions were prepared with distilled water. In a typical three-step synthesis procedures, Sb_2_Se_3_/Sb_2_O_3_@attapulgite composites with 71% of attapulgite mass ratio were synthesized as showed in Fig. S6. K(SbO)C_4_H_4_O_6_·0.5H_2_O (0.332 g) and attapulgite (0.520 g) were dispersed in 45 ml of distilled water under constant stirring for 30 min. Then the precursor was treated with 9 h of sonochemical pretreatment to ensure the system dispersed well, and gradually formed Sb_2_O_3_@attapulgite particles.

Subsequently, 0.064 g of Se powder and 0.061 g of NaBH_4_ were added into 15 ml of distilled water. The solution under continuous stirring until the solution turned to clear colorless and the stirring time was about 15 min. Finally, the above two systems were mixed into a teflon-lined autoclave of 80 ml capacity, sealed and maintained at 180 °C for 10 h. The sample was collected and washed with distilled water for three times, then dried at 80 °C for 6 h. In addition, a set of Sb_2_Se_3_/Sb_2_O_3_@attapulgite composites with different attapulgite mass content was synthesized via controlled the attapulgite added amount and the material preparation parameter information was showed in Table S1. In order to express conveniently, the Sb_2_Se_3_/Sb_2_O_3_@attapulgite composites in the below text without special note indicated that the attapulgite mass was 71%. For comparison, the Sb_2_Se_3_-Sb_2_O_3_-attapulgite material was prepared under the same process as above but without 9 h of sonochemical pretreatment.

### Characterization

The crystalline phases of the sample were characterized by X-ray diffraction (XRD) patterns on a DX-2700 X-ray diffractometer using Cu Kα-radiation. The morphology and element of the products were performed on a TESCAN MIRA3 field emission scanning electron microscope (SEM), which was equipped with an Oxford X-Max 20 energy-dispersion spectrum (EDS) analyzer. Transmission electron microscopy (TEM) and high resolution TEM (HRTEM) were detected by Tecnai G2 F20 microscope equipped with an EDAX data analyzer and operated at 200 KV. X-ray photoelectron spectroscopy (XPS) was taken on a Thermo Fisher Scientific K-Alpha 1063 with Al Kα X-ray radiation source. The binding energy was referred to the C_1s_ peak (binding energy = 284.6 eV). Nitrogen adsorption-desorption isotherms were detected on Micromeritics ASAP 2020 equipment at 77 K. All the samples were dried at 150 °C for 8 h before the measurements. The specific surface area (S_BET_) was calculated by Brunauer-Emmett-Teller (BET) equation, while the micropore surface area (S_micro_), external surface area (S_ext_) and micropore volume (V_micro_) were determined from the isotherms by t-plot methods. The total pore volume (V_total_) was obtained from the adsorbed liquid nitrogen at relative pressure approximately 0.99 and the average pore size (PZ) was calculated from PZ = 4V_total_/S_BET_. The UV–vis diffuse reflectance spectra (UV-vis) were collected on a Cary-100 spectrophotometer over the wavelength range 250–800 nm. The fourier transform infrared analysis (FTIR) was recorded on a Bruker VERTEX-70 spectrometer with KBr pellets, and over the range 4000–400 cm^−1^. The mass content of Sb and Se in Sb_2_Se_3_-Sb_2_O_3_-attapulgite and Sb_2_Se_3_/Sb_2_O_3_@attapulgite composites were detected by inductive coupled plasma emission spectrometer (ICP, Baird PS-6).

### Catalytic activity evaluation

Catalytic test of the as prepared products were performed for the reduction of p-NP to p-AP by excess freshly prepared NaBH_4_. This was a well-known model reaction^29^ to evaluate the catalytic rate of functional materials and the catalytic reaction as Eq. (1). The absorbance maximum (λ_max_) was 317 nm for p-NP in aqueous where the condition was acidic. When adding the excess NaBH_4_ to the p-NP aqueous, the absorbance maximum (λ_max_) shifted to 400 nm because the p-NP ions have produced in the aqueous solution.1


In a typical procedure, 0.5 ml of 0.005 mol/L p-NP solution and 30 ml of 0.033 mol/L NaBH_4_ solution were mixed in a beaker under continuous stirring. The color of the solution immediately turned to bright yellow, which indicated that the p-NP converted to the p-NP ions^67^. Then, 0.02 g of as-synthesis sample was added into the solution. The contents of p-NP ions were determined by 722 s visible spectrophotometer with the absorbance maximum peak at 400 nm.

## Electronic supplementary material

Supporting Information
