# Supplementary material for: Sb2Se3 assembling Sb2O3@ attapulgite as an emerging composites for catalytic hydrogenation of p-nitrophenol
Source: Sci Rep. 2017 Jun 12;7:3281. doi: 10.1038/s41598-017-03281-z (PMC5468295; doi:10.1038/s41598-017-03281-z)
Supplement: Supplementary file 1 — Supporting Information [file 41598_2017_3281_MOESM1_ESM.pdf]

Supporting Information

Sb_2Se_3 assembling Sb_2O_3@ attapulgite as an emerging composites for catalytic hydrogenation of *p*-nitrophenol

Lin Tan[1], Aidong Tang[*1], Yue Zou[1], Mei Long[1], Yi Zhang[2,3], Jin Ouyang[2,3], Jin Chen [*4]

[1] School of Chemistry and Chemical Engineering, Central South University, Changsha 410083, China

[2] Centre for Mineral Materials, School of Minerals Processing and Bioengineering, Central South University, Changsha 410083, China

[3] Hunan Key Laboratory of Mineral Materials and Application, Central South University, Changsha 410083, China

[4] Key Laboratory of Palygorskite Science and Applied Technology of Jiangsu Province, Huaiyin Institute of Technology, Huaian 223003, China

Corresponding authors:: Aidong Tang (email: adtang@csu.edu.cn; tangaidong@126.com) or Jin Chen (email: chenjing6910@163.com)

Table S1

The parameter of the Sb_2Se_3/Sb_2O_3@ATP hybrid composites with different ATP mass amounts.

Sample codes	Antimony potassium tartrate amounts/g	ATP amounts/g	Se powder amounts/g	Sodium borohydride amounts/g	ATP mass content /%	Time /h	Temperature / ℃
a	0.332	1.170	0.064	0.061	85	10	180
b	0.332	0.520	0.064	0.061	71	10	180
c	0.332	0.195	0.064	0.061	48	10	180
d	0.332	0.087	0.064	0.061	29	10	180
e	0.332	0.033	0.064	0.061	14	10	180

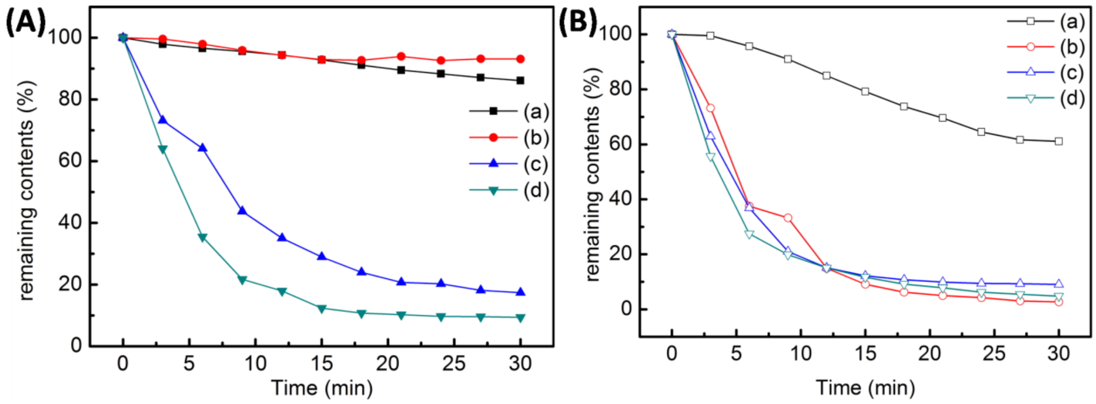

Fig. S1 Catalytic reduction of p-NP in the presence of (A) Sb_2Se_3-Sb_2O_3-ATP and (B) Sb_2Se_3/Sb_2O_3@ATP hybrid composites with different ATP mass amount: (a) 85% ATP, (b) 48% ATP, (c) 29% ATP and (d) 14% ATP.

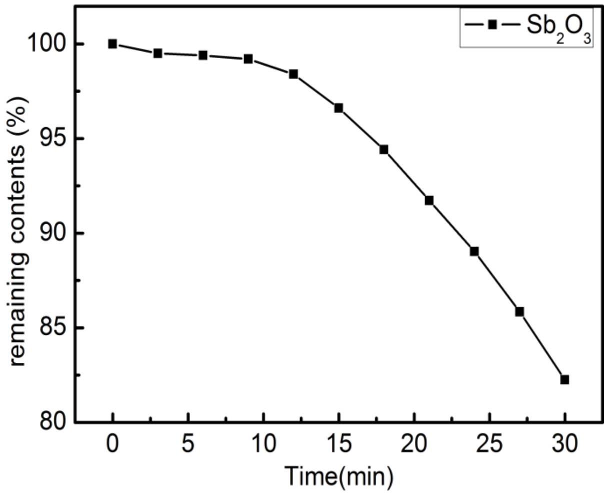

Fig. S2 Plots of p-NP contents versus time in the presence of the Sb_2O_3.

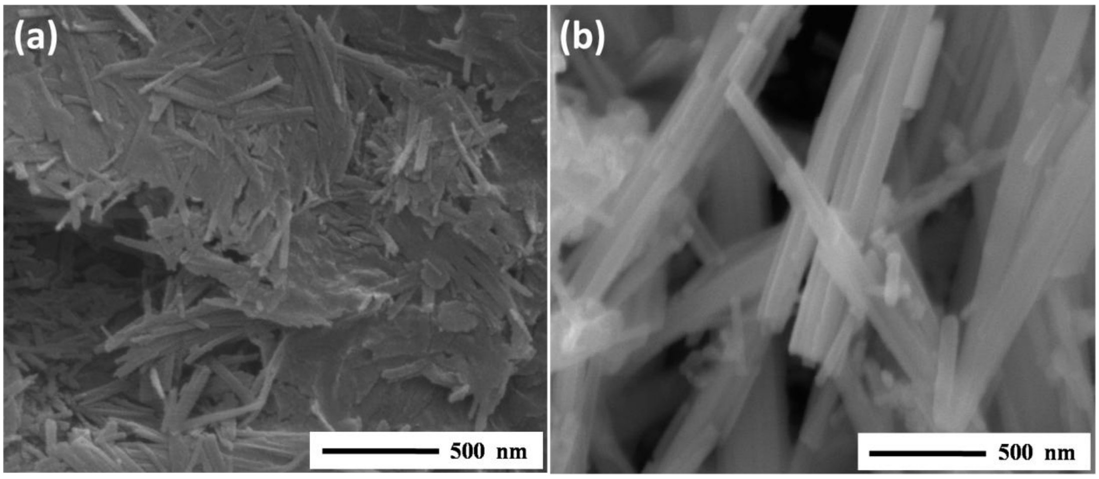

Fig. S3 High magnification SEM images of (a) raw ATP (b) Sb_2Se_3 sample

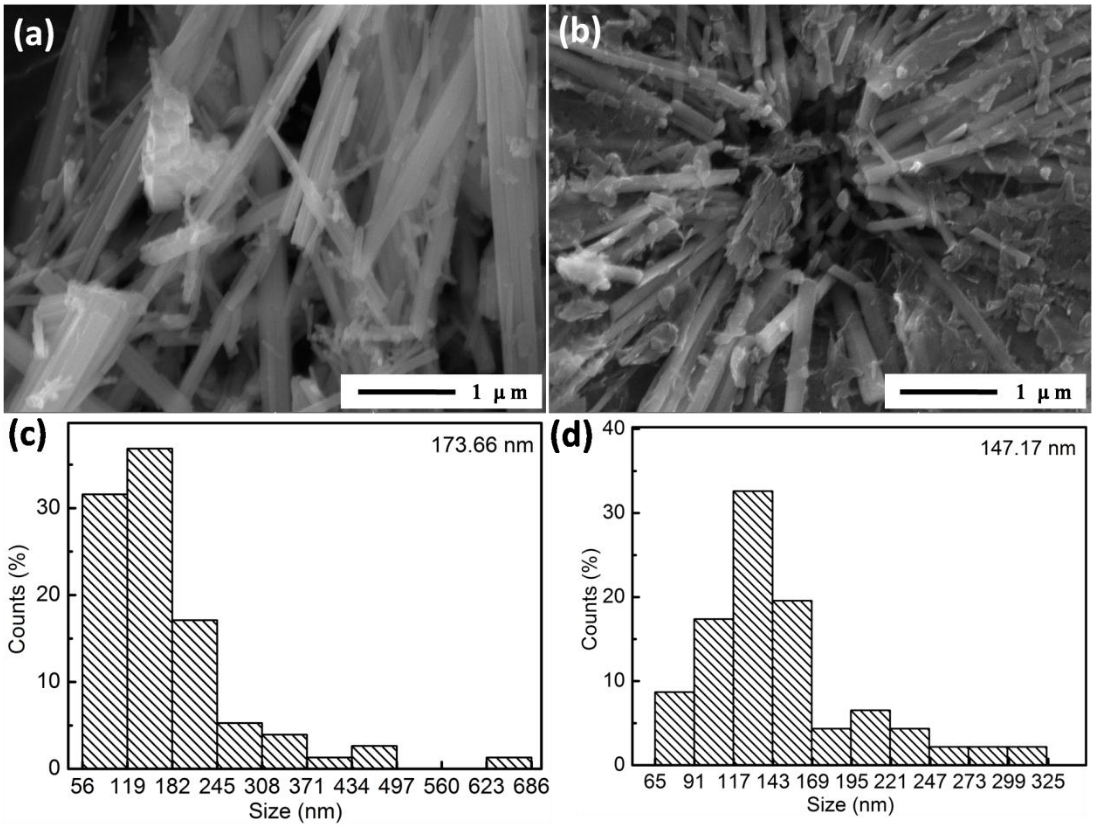

Fig. S4 SEM images of (a) Sb_2Se_3 sample and (b) Sb_2Se_3/Sb_2O_3@ATP; corresponding size distribution diagram of (c) Sb_2Se_3 sample and (d) Sb_2Se_3/Sb_2O_3@ATP.

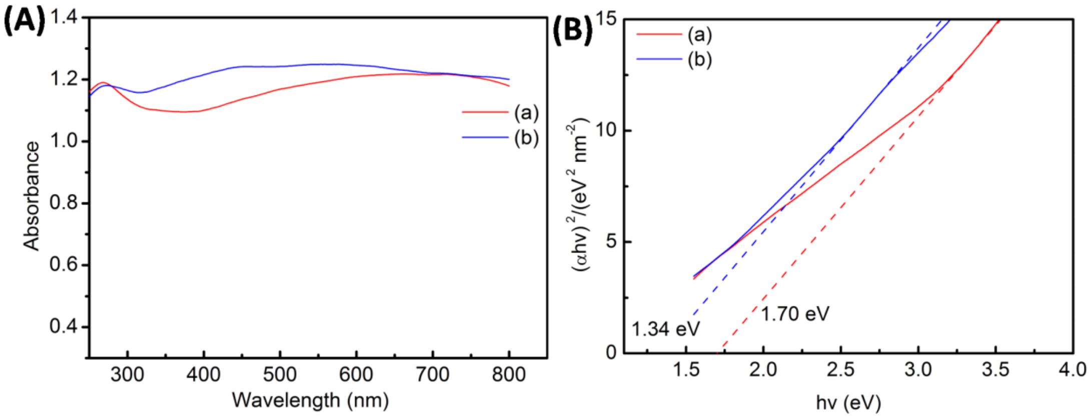

Fig. S5 (A) UV–vis spectra and (B) corresponding $(\alpha h\nu)^2$-(hν) plots of the samples: (a) Sb_2Se_3-Sb_2O_3-ATP and (b) Sb_2Se_3/Sb_2O_3@ATP.

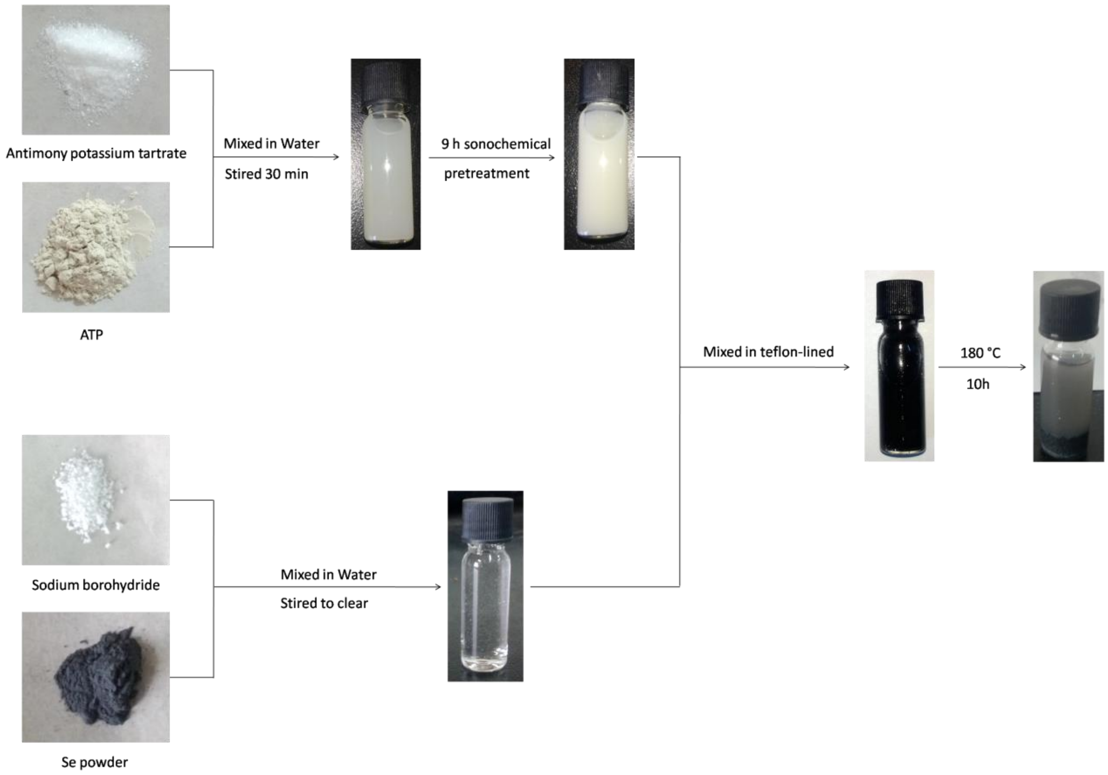

Fig. S6 Fabricated process of Sb_2Se_3/Sb_2O_3@attapulgite composites.
